# Association between prediabetes and the risk of atrial fibrillation: a systematic review and meta-analysis

**DOI:** 10.3389/fendo.2025.1763810

**Published:** 2026-01-19

**Authors:** Yongchao Li, Ju Deng, Li Li

**Affiliations:** 1 Jinan University, Guangzhou, China; 2 Department of Critical Care Rehabilitation, The First People’s Hospital of Chenzhou, Chenzhou, China; 3 Department of Cardiology, Guangzhou Red Cross Hospital, Guangzhou, China

**Keywords:** atrial fibrillation, meta-analysis, prediabetes, risk factors, systematic review

## Abstract

**Background:**

Atrial fibrillation (AF) is the most common sustained cardiac arrhythmia and a major contributor to morbidity and mortality. Although diabetes is a well-established risk factor for AF, the role of prediabetes—a modifiable metabolic condition—remains uncertain. Clarifying this relationship may help identify individuals at risk and guide early preventive strategies.

**Methods:**

We performed a systematic review and meta-analysis of cohort studies identified through PubMed, Embase, and Web of Science databases up to November 21, 2025. Studies reporting adjusted hazard ratios for incident AF in adults with prediabetes compared with normoglycemic controls were included. Pooled estimates were calculated using random-effects models, and heterogeneity was assessed using the I² statistic. Prespecified subgroup analyses explored variations by definition of prediabetes, geographic region, follow-up duration, age, and sex. Publication bias was evaluated using Egger’s and Begg’s tests.

**Results:**

Twelve independent datasets from 11 cohort studies, including over 15 million participants and 277,164 incident AF cases, were analyzed. Prediabetes was associated with a modest but statistically significant increased risk of AF (pooled hazard ratio: 1.20; 95% confidence interval: 1.08–1.35), with substantial heterogeneity. Sensitivity analyses showed consistent results. Subgroup analyses indicated a numerically stronger association in Asian populations than in Europe and North America; this finding should be interpreted cautiously given heterogeneity and limited studies per subgroup. Other subgroup analyses were broadly consistent, and overall evidence of publication bias was limited.

**Conclusion:**

Prediabetes is associated with increased AF risk across diverse populations. Given the observational design, these findings indicate association rather than causation. Early identification and management of prediabetes may provide an opportunity for AF prevention.

**Systematic review registration:**

https://www.crd.york.ac.uk/prospero/, identifier CRD420251233423.

## Introduction

Atrial fibrillation (AF) is the most common sustained cardiac arrhythmia globally, with prevalence increasing markedly with advancing age (1). Large-scale epidemiological projections from Europe estimate that AF prevalence may reach 10%–17% among individuals older than 80 years. With the acceleration of global population aging, the burden of AF is expected to rise further (2). Supporting this trend, the GBD studies have documented a sharp increase in the number of AF and atrial flutter cases over recent decades, from approximately 33.5 million in 2010 to nearly 59.7 million in 2019 (3). Concurrently, AF-related mortality has increased substantially, rising from approximately 117,000 deaths in 1990 to 315,000 deaths in 2019, highlighting the significant public health impact of AF (4). Beyond its epidemiological burden, AF profoundly impairs quality of life and is strongly associated with serious complications, including stroke, heart failure, and cognitive decline, such as dementia, establishing it as a major global health challenge (5–7).

Numerous cardiovascular and metabolic factors have been identified as key risk determinants for AF, including advanced age, male sex, hypertension, coronary artery disease, obesity, and diabetes mellitus (8, 9). Among these, diabetes has been consistently demonstrated to be an independent predictor of AF (10). Recognition of the critical role of diabetes in AF risk has spurred growing interest in whether prediabetes, the early metabolic stage preceding diabetes, may also contribute to the development of AF (11).

Prediabetes represents an intermediate metabolic state characterized by blood glucose levels that are elevated above the normal range but do not meet diagnostic criteria for diabetes. It is commonly defined by impaired fasting glucose (IFG), impaired glucose tolerance (IGT), or mildly elevated glycated hemoglobin (HbA_1_c) (12) Levels. Global data show that the prevalence of prediabetes is rising, now exceeding 30% of the adult population in many countries, and is associated with a substantially increased risk of progression to type 2 diabetes and cardiovascular disease (13). However, current evidence regarding the association between prediabetes and the risk of atrial fibrillation (AF) remains inconsistent. For example, Desai et al. reported that patients with AF and comorbid prediabetes had a higher risk of major adverse cardiovascular and cerebrovascular events, suggesting that prediabetes may negatively influence clinical outcomes in individuals with AF (14). In contrast, Latini et al. found that impaired glucose regulation did not independently predict incident AF, indicating that dysglycemia alone may not confer a clearly elevated AF risk (15). These conflicting findings highlight ongoing uncertainty about whether prediabetes should be considered an independent risk factor for AF.

From both public health and clinical perspectives, clarifying the relationship between prediabetes and AF carries significant implications. Prediabetes is a highly modifiable metabolic condition, and interventions such as lifestyle modification—including dietary changes, increased physical activity, and weight management—as well as targeted pharmacotherapy, can markedly reduce the progression to diabetes and cardiovascular events (16, 17). Therefore, if prediabetes is established as an independent risk factor for AF, early metabolic intervention could serve as a crucial primary prevention strategy to lower the incidence of AF.

Given the current inconsistencies in study findings and the absence of high-quality, quantitative synthesis across varying definitions of prediabetes (i.e., IFG, IGT, and HbA_1_c) and diverse population subgroups, a rigorous systematic review and meta-analysis are warranted. Accordingly, this study aims to synthesize existing evidence from prospective and retrospective cohort studies, providing a comprehensive overview to inform clinical understanding and guide future research.

## Materials and methods

This meta-analysis was conducted in accordance with the guidelines outlined in the Cochrane Handbook for Systematic Reviews of Interventions and the Preferred Reporting Items for Systematic Reviews and Meta-Analysis (PRISMA) guidelines (18, 19). The study protocol was registered with PROSPERO under the registration code CRD420251233423.

### Literature search

To identify studies relevant to the research question, we systematically searched PubMed, Embase, and Web of Science from their inception to November 21, 2025. The search strategy included terms related to prediabetes (“prediabetic state”, “prediabetes”, “impaired fasting glucose”, “impaired glucose tolerance”, IFG, IGT, “glucose intolerance”) and “atrial fibrillation”. Only human studies published as full-length articles in peer-reviewed English journals were considered. When a single study reported more than one independent dataset, each cohort was treated as an independent dataset in the meta-analysis. Reference lists of related articles were also reviewed to capture additional eligible studies. The complete search strategy is provided in **Supplementary Table 1**.

Because the definition of prediabetes varies across clinical guidelines, the diagnostic thresholds used in each included study were recorded. The World Health Organization (WHO) defines IFG as a fasting plasma glucose level of 6.1–6.9 mmol/L and IGT as a 2-h plasma glucose level of 7.8–11.0 mmol/L (20). In contrast, the American Diabetes Association (ADA) defines IFG using a lower threshold of 5.6–6.9 mmol/L or HbA1c levels of 5.7%–6.4% (21). These definitional differences were carefully considered when reviewing individual study criteria and assessing potential sources of heterogeneity.

### Inclusion criteria

Studies were included in this meta-analysis if they met the following criteria: (1) involved adult populations (aged 18 years or older), without specifically excluding individuals with pre-existing cardiovascular diseases or other chronic conditions; (2) clearly defined prediabetes as the exposure, using established diagnostic criteria such as IFG, IGT, or HbA_1_c defined prediabetes; (3) compared individuals with prediabetes to those with normoglycemia; (4) reported the incidence of AF confirmed through electrocardiography, medical records, or validated diagnostic codes; (5) employed either a prospective or retrospective cohort study design; and (6) were published as full-length articles in peer-reviewed English-language journals and reported adjusted effect estimates (hazard ratios) with corresponding 95% confidence intervals.

### Exclusion criteria

Studies were excluded if they met any of the following criteria: (1) involved children or adolescents younger than 18 years of age; (2) focused exclusively on patients with specific diseases (such as myocardial infarction, heart failure, or dialysis populations), rather than general or community-based cohorts; (3) did not clearly define prediabetes or failed to distinguish prediabetes from diabetes or normoglycemia; (4) lacked a normoglycemic comparison group or did not assess the association between prediabetes and incident AF; (5) did not report incident AF or used non-standard or unvalidated methods to ascertain AF; or (6) were reviews, editorials, preclinical studies, conference abstracts, or studies that did not report adjusted effect estimates required for meta-analysis.

### Study selection and data extraction

Yongchao and Ju Deng independently performed study selection and data extraction using a predefined, standardized form. For studies with unclear methodological details, the reviewers contacted the original authors to obtain additional information. Discrepancies between reviewers were resolved through discussion or, when necessary, by consulting the corresponding author (Prof. Li Li) to reach a consensus. Extracted data included the first author’s name, publication year, country or region, participant age, sex distribution, study design, total sample size, diagnostic criteria for prediabetes (including IFG, IGT, or HbA_1_c-defined prediabetes), characteristics of the normoglycemic comparison group, methods used to ascertain incident AF, number of AF cases, duration of follow-up, and covariates adjusted for in the analysis of the association between prediabetes and AF risk.

### Quality assessment

The methodological quality of the included studies was evaluated using the Newcastle–Ottawa Scale (NOS) (22, 23), which assesses three major domains: (1) selection of participants (four items, one star each); (2) comparability of exposed and non-exposed groups (one item, up to two stars); and (3) outcome assessment (three items, one star each). Total scores range from 0 to 9, with studies classified as high quality if they scored ≥7 points, moderate quality if they scored 4–6 points, and low quality if they scored <4 points. Two reviewers independently conducted the quality assessment for each study, and any disagreements were resolved through discussion or consultation with a third senior investigator. The assessment emphasized the adequacy of prediabetes definitions (IFG, IGT, HbA_1_c), methods used to ascertain incident AF, handling of potential confounders, and completeness of follow-up.

### Statistical analysis

Statistical analyses were conducted in accordance with the Cochrane Collaboration guidelines. The association between prediabetes and the risk of AF was quantified using adjusted hazard ratios (HRs) with corresponding 95% confidence intervals (CIs). All effect estimates were log-transformed to stabilize variance, and standard errors were derived from the reported CIs. Heterogeneity across studies was assessed using the Cochrane Q test and the I² statistic, with I² values of 0–25%, 26–50%, and >50% interpreted as indicating low, moderate, and substantial heterogeneity, respectively (24). A fixed-effects model was applied when heterogeneity was low (*P* > 0.1 and I² < 50%), whereas a random-effects model was used in the presence of moderate or substantial heterogeneity.

Prespecified subgroup analyses were performed based on prediabetes diagnostic criteria (i.e., ADA- or WHO-defined IFG, IGT, or elevated HbA1c), geographic region, follow-up duration, mean age of participants, and proportion of male participants. Sensitivity analyses were conducted by sequentially omitting each study to assess the robustness of the pooled estimates. Publication bias was evaluated using Begg’s and Egger’s (25, 26). When potential publication bias was detected, the nonparametric trim-and-fill method was employed to estimate its influence on the overall effect size. All statistical analyses were performed using RevMan software (version 5.4; Cochrane Collaboration, Oxford, UK) and Stata software (version 14.0; Stata Corporation, College Station, TX). Two-tailed *P*-values < 0.05 were considered statistically significant.

## Results

### Basic characteristics and quality assessment

**Figure 1:**
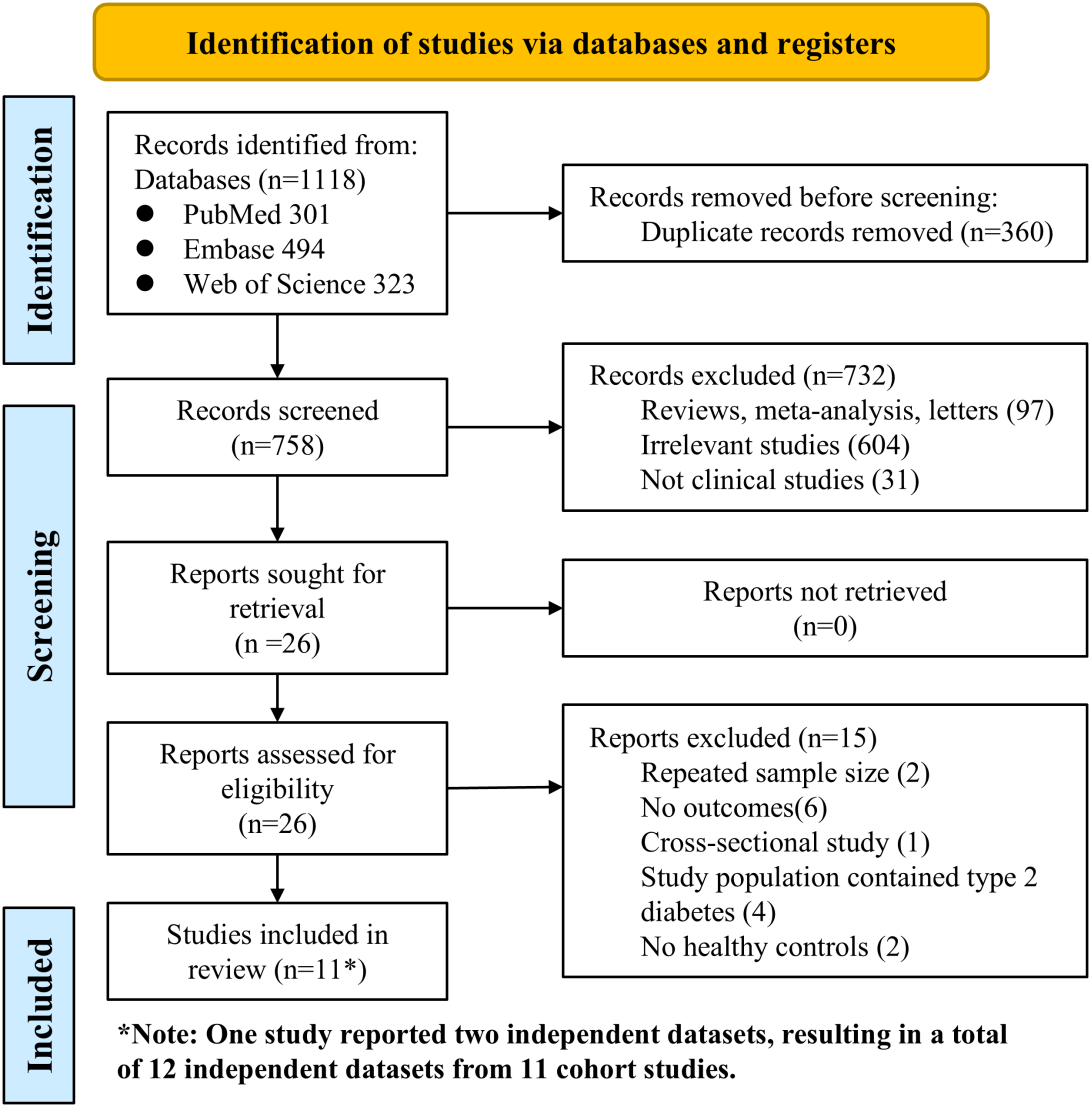
PRISMA flowchart presenting the study selection process for this systematic review and meta-analysis.

The systematic search yielded 1,118 records from PubMed, Embase, and Web of Science. After the removal of duplicates, 758 articles remained. Title and abstract screening led to the exclusion of 732 records that did not meet the eligibility criteria. The full texts of 26 studies were assessed for eligibility, of which 15 were excluded. One study provided two independent cohorts from Korea and the United Kingdom, which were treated as separate datasets in the meta-analysis (27). Resulting in 12 independent datasets from 11 cohort studies included in the quantitative synthesis (27–37). The study selection process is illustrated in the PRISMA flow diagram (**Figure 1**).

Among the included studies, seven were conducted in Asia (three in Korea, two in China, and two in Japan), while five were from Europe and North America (two in Sweden and one each from the United Kingdom, the United States, and Denmark). The combined study population comprised 15,676,887 participants, with a mean age of 53.1 years (range: 39.7–67.0 years). Of these, 2,976,805 individuals (19%) were classified as having prediabetes according to either WHO or ADA diagnostic criteria. The median follow-up duration was 9.4 years (range: 3.2–19.1 years). Incident AF was identified using ICD-9 or ICD-10 codes. Three studies included atrial flutter in the atrial fibrillation definition (28, 30, 31), while the remaining studies reported atrial fibrillation alone, resulting in 277,164 documented cases.

All studies adjusted for age and sex in their multivariable models. Additional covariates varied across studies but commonly included smoking status, alcohol consumption, body mass index, blood pressure, antihypertensive medication use, and lipid levels. Several studies also accounted for socioeconomic indicators and major comorbidities such as hypertension, dyslipidemia, diabetes, or prior cardiovascular disease. Detailed characteristics of the included studies are provided in **Table 1**.

**Table 1:** Characteristics of the included studies.

Study	Country	Design	Sample size	Mean age (years)	Male (%)	Prediabetes definition	No. of people with PrD	AF diagnosis	No. of AF	Mean follow-up duration(years)	Variables adjusted	Relative risk adjusted HR (95%CI)
C. Johansson 2023 (28)	Sweden	RC	88889	49.0	48.2	IFG (WHO)/IGT (WHO)	7462/2970	ICD-10 code I48 or ICD-9 code 427.3	4948	10.2	Sex, age, BMI, systolic blood pressure, antihypertensive drugs, total cholesterol, alcohol, smoking, education level, marital status, physical activity	1.03 (0.93, 1.14)/0.86 (0.74, 1.01)
H. Kaneko 2021 (29)	Japan	RC	1180062	39.7	57	IFG (WHO)	32451	ICD-10 codes	2828	3.3	Age, sex, obesity, high waist circumference, hypertension, dyslipidemia, cigarette smoking, alcohol drinking	1.13 (0.93, 1.36)
Jung‑Chi Hsu 2023 (30)	China	RC	28618	64.5	46.9	IFG or IGT or HbA1c (ADA)	14309	ICD-10 code I48	654	3.93	Age, sex, BMI, HTN, hyperlipidemia, gout, CAD, COPD, PAOD, eGFR, history of HF, history of TIA/ischemic stroke, NT-pro-BNP, LVEF, LA size, LV mass	1.24 (1.11, 1.39)
Juntae Kim 2023a (27)	Korea	RC	176937	52.0	53.1	IFG (ADA)	40453	ICD-10 code I48	2346	7.4	Age, sex, smoking, alcohol intake, economic status	1.14 (1.04, 1.25)
Juntae Kim 2023b (27)	UK	PC	167946	54.4	37.2	IFG (ADA)	14365	ICD-10 code I48	5314	11.8	Age, sex, smoking, alcohol intake, economic status	1.1 (1.01, 1.2)
R. R. Huxley 2012 (31)	USA	PC	13025	57.0	44.1	IFG or HbA1c (ADA)	6693	ICD-9 codes 427.31 or 427.32	1311	14.5	Age, race, sex, center, education, income, smoking status, prevalent CHD, systolic blood pressure, hypertensive medications and body mass index	0.96 (0.84, 1.1)
Rasmus Rørth 2023 (32)	Denmark	RC	354807	63.0	47	HbA1c (WHO)	27754	ICD-10 codes	19649	12	Age, sex, year of baseline, ischemic heart disease, cancer, renal disease, COPD, hypertension	1.12 (1.08, 1.16)
Sean S. Lee 2017 (33)	Korea	RC	227102	≥20	48.9	IFG (ADA)/IFG (WHO)	40317/ 11900	ICD-10 code I48	1470	7.7	Age, gender, smoking status, alcohol intake frequency	1.16 (1.03, 1.31)/1.21 (1.01, 1.45)
Viktor Lind 2021 (34)	Sweden	PC	294057	47.0	53.9	IFG (WHO)	9682	ICD codes	28233	19.1	Attained age (timescale), sex, total cholesterol, triglycerides, socioeconomic index	1.19 (1.13, 1.26)
Xinyi Yu 2025 (35)	China	PC	11663	67.0	49.2	IFG and/or HbA1c (ADA)	4264	ICD-10 code I48	1343	11.1	Age, sex, smoking, alcohol consumption, regular physical activity, SBP, DBP, TG, LDL-c, HDL-c.	2.2 (1.94, 2.51)
Yun Gi Kim 2019 (36)	Korea	RC	9797418	47.0	54.7	IFG (ADA)	2215482	ICD-10 codes	196136	8.18	Age, sex, smoking, alcohol consumption, physical activity, income level, diabetes, hypertension, dyslipidemia, etc.	1.464 (1.45, 1.48)
Yuta Suzuki 2023 (37)	Japan	RC	3336363	43.0	57.2	IFG (ADA)	563573	ICD-10 codes	12932	3.2	Age, sex, body mass index, dyslipidemia, cigarette smoking, alcohol consumption	1.08 (1.02, 1.15)

RC, Retrospective Cohort Study; PC, Prospective cohort study; AF, Atrial fibrillation; IFG, Impaired fasting glucose; IGT, Impaired glucose tolerance; HbA_1_c, Glycated hemoglobin; WHO, World Health Organization; ADA, The American Diabetes Association; HR, Hazard ratios; CIs, Confidence intervals; BMI, Body Mass Index.

All 12 independent datasets from 11 cohort studies achieved NOS scores ranging from 7 to 9, indicating high methodological quality (**Table 2**).

**Table 2:** Newcastle–Ottawa score for risk-of-bias assessment of the included studies.

Study	Representativeness of the exposed cohort	Selection of the non-exposed cohort	Ascertainment of exposure	Outcome not present at baseline	Control for age and sex	Control for other confounding factors	Assessment of outcome	Enough long follow-up duration	Adequacy of follow-up of cohort	Total
C. Johansson 2023 (28)	1	1	1	1	1	1	1	1	1	9
H. Kaneko 2021 (29)	1	1	1	1	1	0	1	0	1	7
Jung‑Chi Hsu 2023 (30)	1	1	1	1	1	1	1	0	1	8
Juntae Kim 2023a (27)	1	1	1	1	1	0	1	1	1	8
Juntae Kim 2023b (27)	1	1	1	1	1	0	1	1	1	8
R. R. Huxley 2012 (31)	1	1	1	1	1	1	1	1	0	8
Rasmus Rørth 2023 (32)	1	1	1	1	1	1	1	1	0	8
Sean S. Lee 2017 (33)	1	1	1	1	1	1	1	1	1	9
Viktor Lind 2021 (34)	1	1	1	1	1	0	1	1	1	8
Xinyi Yu 2025 (35)	1	1	1	1	1	0	1	1	1	8
Yun Gi Kim 2019 (36)	1	1	1	1	1	1	1	1	1	9
Yuta Suzuki 2023 (37)	1	1	1	1	1	1	1	0	1	8

### Sensitivity analysis

**Figure 2:**
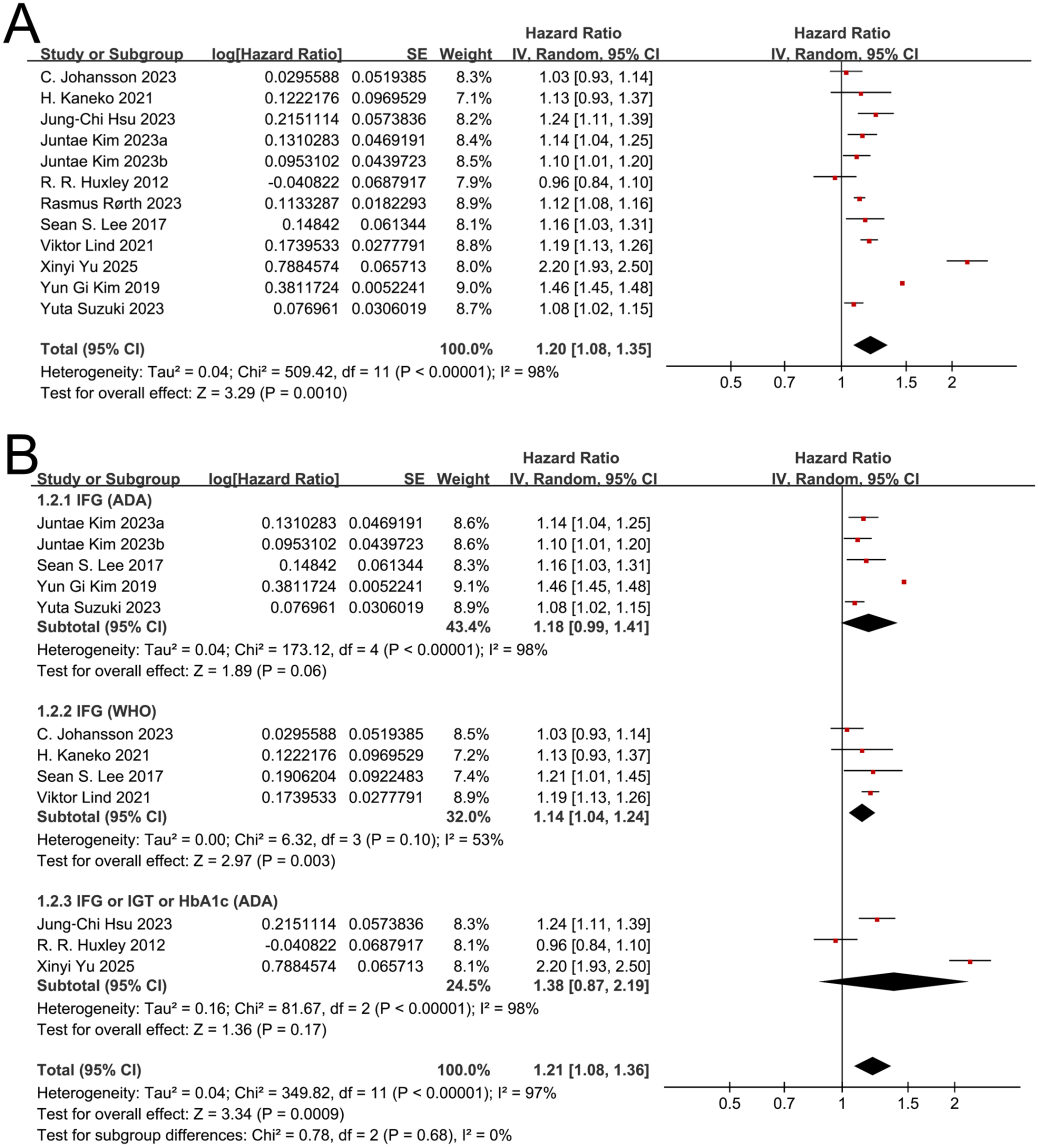
Forest plots for the association between prediabetes and the risk of atrial fibrillation. **(A)** Overall meta-analysis; **(B)** Subgroup analysis according to the diagnosis of prediabetes.

The pooled analysis of 12 independent datasets from 11 cohort studies demonstrated a significant association between prediabetes and the risk of AF. Given the presence of substantial heterogeneity (I² = 98%), a random-effects model was applied, yielding a combined HR of 1.20 (95% CI 1.08–1.35; *P* = 0.0010) (**Figure 2A**). Most studies reported point estimates greater than 1.00, and nine demonstrated statistically significant associations (27, 30, 32–37). Three studies had confidence intervals that crossed unity (28, 29, 31). The largest effect size was reported by Xinyi Yu et al. (HR = 2.20) (35), while the most precise estimate, as indicated by the narrowest CI, was observed in the study by Yun Gi Kim et al. (HR = 1.46) (36).

Sensitivity analyses, conducted by sequentially omitting each study, yielded consistent results (all *P* < 0.05), confirming the robustness of the overall effect estimate. However, heterogeneity remained high (I² > 75%), indicating that no single study accounted for the observed heterogeneity.

### Subgroup analyses

#### Diagnosis of prediabetes

Subgroup analyses were conducted based on the diagnostic criteria used to define prediabetes. These criteria were categorized as ADA-defined IFG, WHO-defined IFG, and a combined ADA-based definition that included IFG, IGT, or elevated HbA1c. Sean S. Lee et. Al (33) contributed separate effect estimates for both ADA and WHO definitions of IFG.

In the subgroup based on ADA-defined IFG, the pooled estimate demonstrated a non-significant trend toward increased AF risk (HR 1.18; 95% CI: 0.99–1.41; *P* = 0.06), with substantial heterogeneity (I² = 98%). For WHO-defined IFG, the association reached statistical significance (HR 1.14; 95% CI: 1.04–1.24; *P =* 0.003), with moderate heterogeneity (I²= 53%). In the subgroup combining ADA-defined IFG, IGT, or elevated HbA1c, the pooled HR was 1.38 (95% CI: 0.87–2.19; *P =* 0.17), with substantial heterogeneity (I² = 98%) (**Figure 2B**). Tests for subgroup differences were not significant (Chi² = 0.78; *P =* 0.68; I² = 0%), indicating that no reliable distinction can be made between diagnostic definitions.

#### Follow-up duration

**Figure 3:**
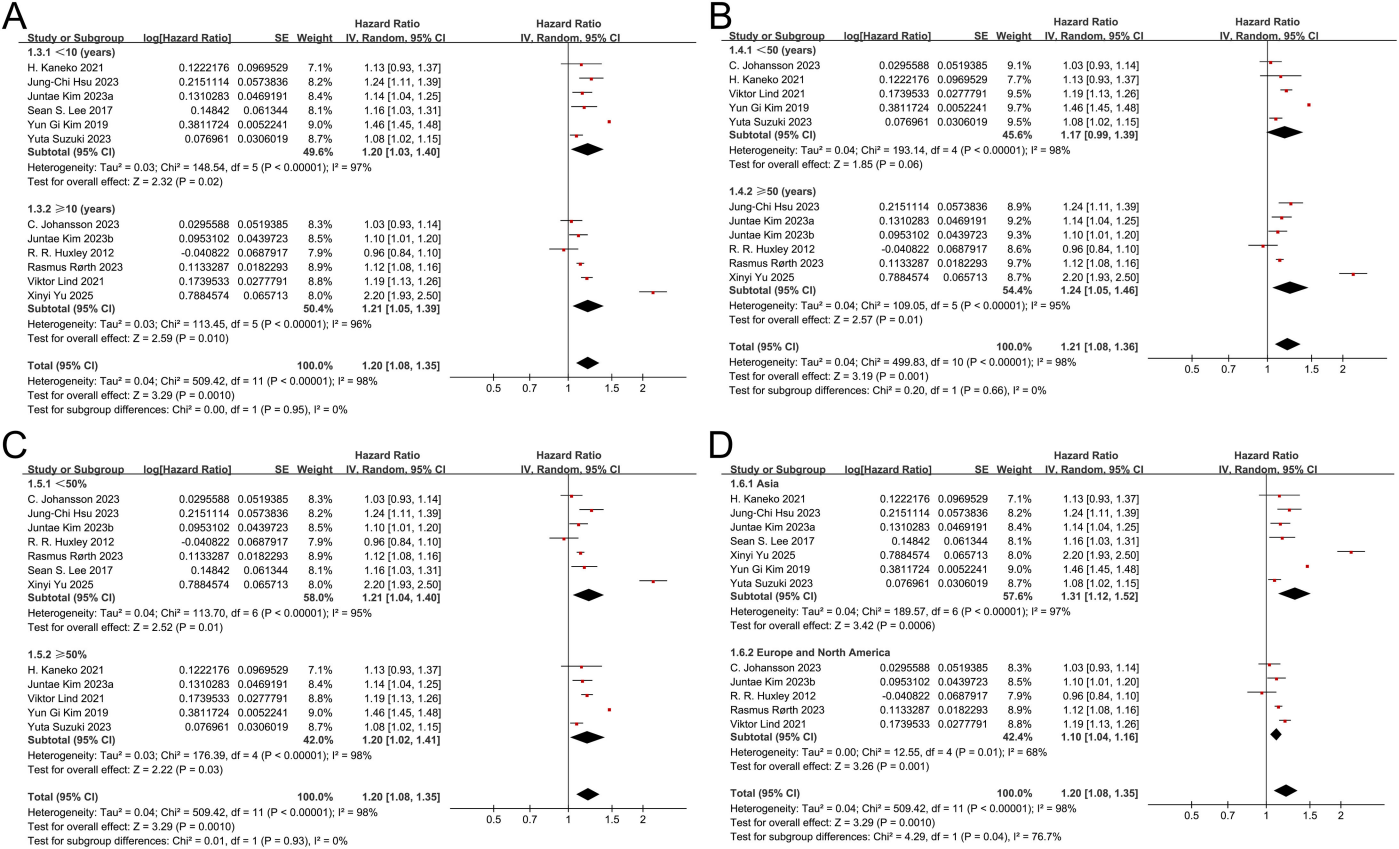
Forest plots for the subgroup analyses of prediabetes and the risk of atrial fibrillation. **(A)** subgroup analysis according to the follow-up duration; **(B)** subgroup analysis according to the mean age of the participants; **(C)** subgroup analysis according to the proportion of men; **(D)** subgroup analysis according to the geographic region.

Subgroup analyses were also conducted based on follow-up duration, categorized as <10 years and ≥10 years (**Figure 3A**). In studies with follow-up <10 years, prediabetes was associated with an increased risk of AF (HR 1.20; 95% CI: 1.03–1.40; *P* = 0.02), with substantial heterogeneity (I² = 97%). A similar association was observed in studies with follow-up ≥10 years (HR 1.21; 95% CI: 1.05–1.39; *P =* 0.010), also accompanied by substantial heterogeneity (I² = 96%). The test for subgroup differences was not statistically significant (Chi² = 0.00; *P =* 0.95; I² = 0%), indicating that follow-up duration did not significantly modify the relationship between prediabetes and incident AF.

#### Mean age of the participants

Subgroup analyses were performed based on the mean age of participants, stratified as <50 years and ≥50 years (**Figure 3B**). In studies with a mean age ≥50 years, prediabetes was significantly associated with an increased risk of AF (HR 1.24; 95% CI 1.05–1.46; *P* = 0.01), accompanied by substantial heterogeneity (I² = 95%). A similar trend was observed in studies with a mean age <50 years (HR 1.17; 95% CI 0.99–1.39), with substantial heterogeneity (I² = 98%), although the association did not reach statistical significance (*P* = 0.06). The test for subgroup differences was not significant (*P* = 0.66), suggesting that the association between prediabetes and AF was not meaningfully modified by the average age of the study population.

### Proportion of male participants

Subgroup analyses were also conducted according to the proportion of male participants in each study (**Figure 3C**). In studies in which men comprised less than 50% of the population, prediabetes was significantly associated with an increased risk of AF (HR 1.21; 95% CI 1.04–1.40; *P =* 0.01), with substantial heterogeneity (I² = 95%). A comparable association was observed in studies with more than 50% male participants (HR 1.20; 95% CI 1.02–1.41; *P =* 0.03), with substantial heterogeneity (I² = 98%). The pooled estimates were consistent across the two subgroups, and the test for subgroup differences was not statistically significant (*P =* 0.93), indicating that the proportion of male participants did not materially affect the association between prediabetes and AF.

#### Geographic region

We conducted a subgroup analysis based on the geographic region of the included studies (**Figure 3D**). In studies involving Asian populations, prediabetes was significantly associated with an increased risk of AF (HR = 1.31; 95% CI: 1.12–1.52, *P =* 0.0006), accompanied by substantial heterogeneity (I² = 97%). In contrast, studies conducted in Europe and North America also demonstrated a significant association, although with a smaller effect size (HR = 1.10; 95% CI: 1.04–1.16, *P =* 0.001) and moderate heterogeneity (I² = 68%). The between-subgroup difference was statistically significant (*P =* 0.04). However, given the limited number of studies in each subgroup and substantial heterogeneity, these findings should be interpreted cautiously.

### Publication bias

**Figure 4:**
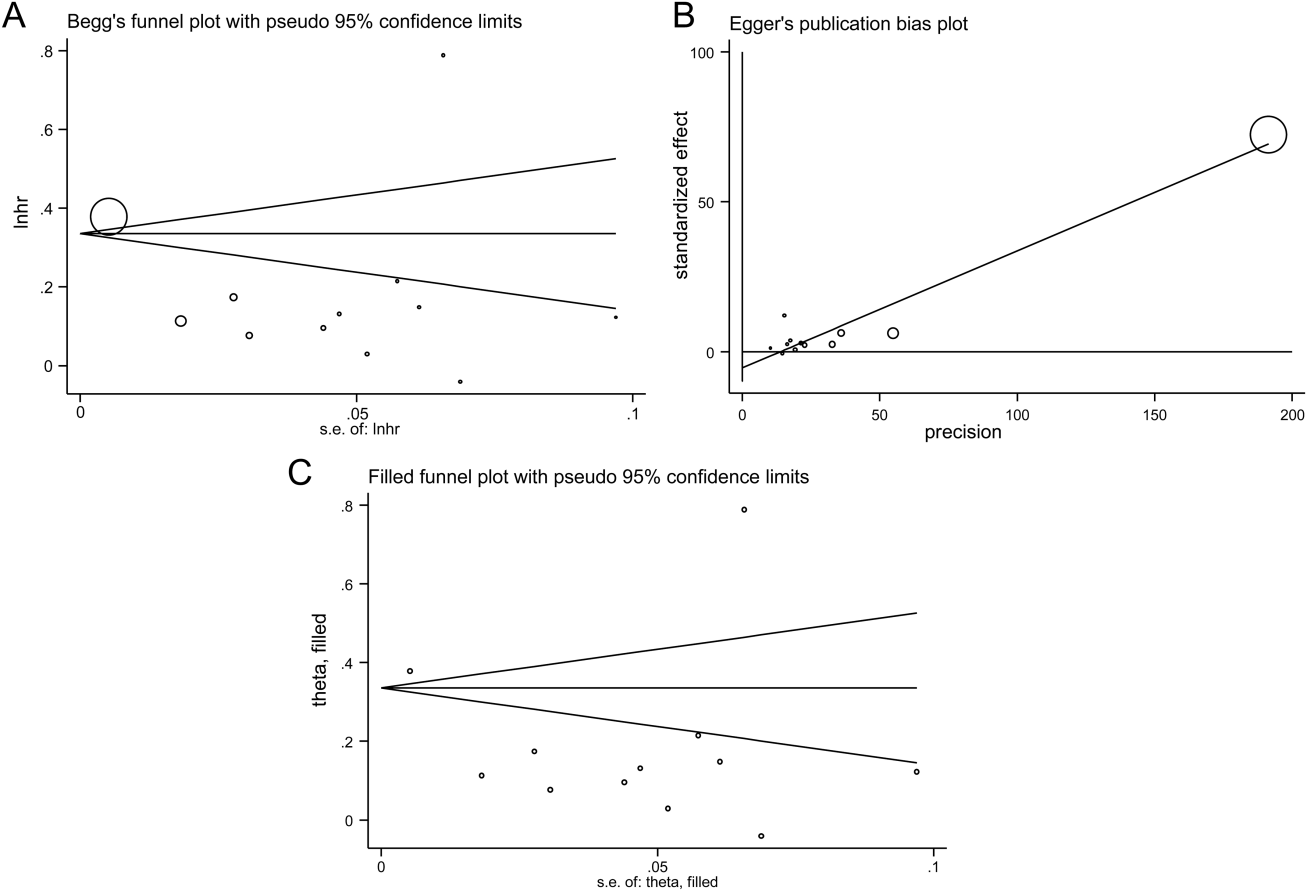
Assessment of publication bias in the meta-analysis of prediabetes and incident atrial fibrillation. **(A)** Begg’s test; **(B)** Egger’s test; **(C)** Funnel plot adjusted using the non-parametric trim-and-fill.

Begg’s test showed no significant evidence of publication bias (*P* = 0.131) (**Figure 4A**), whereas Egger’s test indicated potential bias (bias coefficient = -5.39, *P* = 0.021) (**Figure 4B**). This discordance may reflect the higher sensitivity of Egger’s test to effect size distribution in the presence of substantial heterogeneity. The non-parametric trim-and-fill method suggested no studies required imputation, and the adjusted pooled HR remained unchanged (random-effects model: HR = 1.204; 95% CI: 1.079–1.344, **Figure 4C**). Thus, although possible bias cannot be entirely excluded, its impact on the pooled estimate appears minimal.

## Discussion

In this comprehensive meta-analysis involving more than 15 million individuals, we provide consistent epidemiological evidence that prediabetes is independently associated with an increased risk of incident AF. A pooled HR of approximately 1.20, derived from 12 independent datasets with a median follow-up of nearly 10 years, highlights that even subdiabetic dysglycemia may contribute to arrhythmogenesis. Notably, this association remained consistent in sensitivity analyses and persisted across various subgroup settings, supporting the robustness of the finding. Although statistically significant, the observed effect size is modest and should be interpreted in the context of substantial between-study heterogeneity.

Several early metabolic disturbances associated with prediabetes may explain its link to AF. Mild dysglycemia is frequently accompanied by low-grade inflammation and autonomic imbalance, both of which can initiate structural and electrophysiological changes in the atria (38, 39). Insulin resistance (IR) appears to be central to this pathophysiological process. Data from the Framingham Heart Study have shown that elevated IR predicts incident AF independently of traditional risk factors (40). Experimental studies further suggest that IR promotes oxidative stress, inflammatory and fibrotic signaling, and abnormalities in calcium handling, all of which contribute to atrial remodeling (41). Clinical comorbidities commonly accompanying prediabetes, such as hypertension and obesity, may further amplify its pro-arrhythmic effects. Evidence from animal models supports this notion, demonstrating that diet-induced IR leads to increased atrial fibrosis, heightened oxidative stress, and greater electrophysiological instability (42). These findings suggest that adverse cardiac remodeling may begin early along the glycemic continuum, highlighting the clinical importance of metabolic perturbations in prediabetes for arrhythmia development.

Our subgroup analyses reveal nuances that should be interpreted cautiously. The association between prediabetes and AF was generally consistent across definitions (ADA vs. WHO, glucose-based vs. HbA1c), age groups, sex distributions, and follow-up durations. Methodological and population-level differences across studies may partly explain the observed heterogeneity. The stronger association observed in Asian populations is exploratory, likely reflecting ethnic differences in metabolic risk at lower body mass indices, but should not be considered definitive (43, 44). Similarly, the numerically weaker association for ADA-defined IFG may reflect the lower fasting glucose threshold capturing milder dysglycemia (45); however, no significant subgroup differences were detected.

Given that prediabetes is a potentially modifiable condition, these findings suggest that early interventions, such as lifestyle modification or pharmacotherapy with insulin-sensitizing agents, may help reduce the risk of both diabetes progression and AF onset (46, 47). The results also raise potential implications for clinical practice, including considering prediabetes in AF risk stratification and the value of metabolic risk assessment in AF prevention. However, whether such interventions can effectively lower AF risk remains to be confirmed. Future studies should specifically evaluate the impact of targeted interventions in prediabetic individuals on AF incidence.

Nevertheless, several limitations merit consideration. First, all included studies were observational, so the findings reflect associations rather than causation. Residual confounding cannot be excluded, and unmeasured factors such as physical activity, diet, sleep disorders, socioeconomic status, and subclinical inflammation may have influenced the results. Second, substantial heterogeneity was observed across studies, likely due to differences in prediabetes definitions (ADA vs. WHO; glucose-based vs. HbA1c-based), outcome ascertainment (ECG/clinical records vs. administrative ICD codes), and whether atrial flutter was included in the atrial fibrillation definition. Third, variation in study populations—age distribution, cardiometabolic comorbidities, and regional risk profiles—may have contributed to heterogeneity. Finally, although trim-and-fill analysis suggested minimal publication bias, asymmetry detected by Egger’s test likely reflects its sensitivity in the context of high heterogeneity, with limited impact on overall estimates.

Future research should aim to elucidate the mechanisms through which metabolic abnormalities in prediabetes contribute to atrial remodeling, with particular focus on the potential roles of fibrosis, autonomic dysfunction, and neurohormonal activation. Prospective studies incorporating continuous measures of glucose metabolism and insulin sensitivity, alongside advanced cardiac imaging modalities such as echocardiography or cardiac magnetic resonance imaging, would provide valuable insights into the progression from prediabetes to AF. Additionally, randomized controlled trials are warranted to assess whether interventions targeting prediabetes—such as lifestyle modification or pharmacological therapy—can effectively reduce the risk of AF development.

## Conclusion

This meta-analysis provides evidence that prediabetes is associated with a modest but statistically significant increase in AF risk across diverse populations. Given the observational nature of the included studies, these findings indicate an association rather than a causal relationship. The association was generally consistent across different definitions of prediabetes, follow-up durations, age and sex subgroups, and geographic regions, with a potentially stronger effect in Asian cohorts. These findings support the characterization of prediabetes as a clinical and biologically relevant metabolic risk factor for AF and highlight the importance of early metabolic risk stratification and intervention. Future studies should further investigate the underlying mechanisms and evaluate whether lifestyle or pharmacological interventions during the prediabetic stage can reduce AF incidence.

## Data Availability

The original contributions presented in the study are included in the article/**Supplementary Material**. Further inquiries can be directed to the corresponding author.
