# Supplementary material for: Association between prediabetes and the risk of atrial fibrillation: a systematic review and meta-analysis
Source: Front Endocrinol (Lausanne). 2026 Jan 19;16:1763810. doi: 10.3389/fendo.2025.1763810 (PMC12861884; doi:10.3389/fendo.2025.1763810)
Supplement: Supplementary file 1 [file Table1.docx]

**Supplementary Table 1** The search strategy of each database

| **Database** | **Search strategy** | **Results** |
| --- | --- | --- |
| **PubMed** | #1 "Prediabetic State"[Mesh] OR prediabetes[tiab] OR pre-diabetes[tiab] OR prediabetic[tiab] OR pre-diabetic[tiab] OR "impaired fasting glucose"[tiab] OR "impaired glucose tolerance"[tiab] OR IFG[tiab] OR IGT[tiab] OR "glucose intolerance"[tiab] OR hyperglycemia[tiab] OR "borderline diabetes"[tiab] OR "higher risk of diabetes"[tiab] OR "high risk of diabetes"[tiab] OR "high normal blood glucose"[tiab] | 106597 |
|  | #2 "Atrial Fibrillation"[Mesh] OR "atrial fibrillation"[tiab] OR "auricular fibrillation"[tiab] | 124263 |
|  | #3 #1 and #2 | **301** |
| **Embase** | #1 'prediabetic state'/exp OR prediabetes:ti,ab OR 'pre-diabetes':ti,ab OR prediabetic:ti,ab OR 'pre-diabetic':ti,ab OR 'impaired fasting glucose':ti,ab OR 'impaired glucose tolerance':ti,ab OR ifg:ti,ab OR igt:ti,ab OR 'glucose intolerance':ti,ab OR hyperglycemia:ti,ab OR 'borderline diabetes':ti,ab OR 'higher risk of diabetes':ti,ab OR 'high risk of diabetes':ti,ab OR 'high normal blood glucose':ti,ab | 172662 |
|  | #2 'atrial fibrillation'/exp OR 'atrial fibrillation':ti,ab OR 'auricular fibrillation':ti,ab | 280226 |
|  | #3 #1 AND #2 | 1071 |
|  | #4 #1 AND #2 AND ('article'/it OR 'article in press'/it OR 'clinical trial'/it) AND [english]/lim AND [humans]/lim | **494** |
| **Web of Science** | TS =((prediabetes OR "pre-diabetes" OR prediabetic OR "pre-diabetic" OR "impaired fasting glucose" OR "impaired glucose tolerance" OR IFG OR IGT OR "glucose intolerance" OR hyperglycemia OR "borderline diabetes" OR "higher risk of diabetes" OR "high risk of diabetes" OR "high normal blood glucose") AND ("atrial fibrillation" OR "auricular fibrillation"))  Refined By: Document Types: Article. Languages: English. | **323** |
